# SAGDTI: self-attention and graph neural network with multiple information representations for the prediction of drug–target interactions

**DOI:** 10.1093/bioadv/vbad116

**Published:** 2023-08-26

**Authors:** Xiaokun Li, Qiang Yang, Gongning Luo, Long Xu, Weihe Dong, Wei Wang, Suyu Dong, Kuanquan Wang, Ping Xuan, Xin Gao

**Affiliations:** School of Computer Science and Technology, Heilongjiang University, Harbin 150080, China; Postdoctoral Program of Heilongjiang Hengxun Technology Co., Ltd., Harbin 150090, China; School of Computer Science and Technology, Heilongjiang University, Harbin 150080, China; Postdoctoral Program of Heilongjiang Hengxun Technology Co., Ltd., Harbin 150090, China; School of Computer Science and Technology, Harbin Institute of Technology, Harbin 150001, China; School of Computer Science and Technology, Heilongjiang University, Harbin 150080, China; Postdoctoral Program of Heilongjiang Hengxun Technology Co., Ltd., Harbin 150090, China; Postdoctoral Program of Heilongjiang Hengxun Technology Co., Ltd., Harbin 150090, China; College of Computer and Control Engineering, Northeast Forestry University, Harbin 150040, China; School of Computer Science and Technology, Harbin Institute of Technology, Harbin 150001, China; College of Computer and Control Engineering, Northeast Forestry University, Harbin 150040, China; School of Computer Science and Technology, Harbin Institute of Technology, Harbin 150001, China; School of Computer Science and Technology, Heilongjiang University, Harbin 150080, China; Department of Computer Science, School of Engineering, Shantou University, Shantou 515063, China; Computer, Electrical and Mathematical Sciences & Engineering Division, King Abdullah University of Science and Technology, 4700 KAUST, Thuwal 23955, Saudi Arabia

## Abstract

**Motivation:**

Accurate identification of target proteins that interact with drugs is a vital step *in silico*, which can significantly foster the development of drug repurposing and drug discovery. In recent years, numerous deep learning-based methods have been introduced to treat drug–target interaction (DTI) prediction as a classification task. The output of this task is binary identification suggesting the absence or presence of interactions. However, existing studies often (i) neglect the unique molecular attributes when embedding drugs and proteins, and (ii) determine the interaction of drug–target pairs without considering biological interaction information.

**Results:**

In this study, we propose an end-to-end attention-derived method based on the self-attention mechanism and graph neural network, termed SAGDTI. The aim of this method is to overcome the aforementioned drawbacks in the identification of DTI. SAGDTI is the first method to sufficiently consider the unique molecular attribute representations for both drugs and targets in the input form of the SMILES sequences and three-dimensional structure graphs. In addition, our method aggregates the feature attributes of biological information between drugs and targets through multi-scale topologies and diverse connections. Experimental results illustrate that SAGDTI outperforms existing prediction models, which benefit from the unique molecular attributes embedded by atom-level attention and biological interaction information representation aggregated by node-level attention. Moreover, a case study on severe acute respiratory syndrome coronavirus 2 (SARS-CoV-2) shows that our model is a powerful tool for identifying DTIs in real life.

**Availability and implementation:**

The data and codes underlying this article are available in Github at https://github.com/lixiaokun2020/SAGDTI.

## 1 Introduction

Research on drug–target interaction (DTI) prediction is a crucial step in the repurposing of current drugs and the discovery of new drugs (Ashburn and Thor 2004, Rifaioglu *et al.* 2019, Zheng *et al.* 2020). Accurate identification of potential DTI greatly reduces the requirement of costly and time-consuming high-throughput screening (da Silva Rocha et al. 2019, Bagherian *et al.* 2021). However, it is impractical to rapidly distinguish every possible compound-target pair because of the large-scale chemical space of molecular compounds, proteins, and protein–ligand complexes (Maia *et al.* 2020, Zhao *et al.* 2022). Based on this observation, various computational methods have been introduced to identify potential DTI. In the incipient stage of using computational approaches for DTI identification, molecular simulation, and molecular docking are the mainstream methods (Csermely *et al.* 2013, Śledź and Caflisch 2018). These methods strongly depend on the three-dimensional (3D) structural information of proteins. However, the performance of these structure-based methods is limited due to the inadequate spatial structure for many proteins. In the past decade, machine learning-based approaches were introduced to overcome some of these difficulties in the process of identifying DTI. For example, Ezzat *et al.* (2017) establish a matrix factorization framework with k-nearest known neighbors for DTI prediction that incorporates both drug and target similarities. Conventional shallow machine learning-based methods, such as NRLMF (Liu *et al.* 2016) and KronRLS (Pahikkala *et al.* 2015), only considered the similarity features of drug–target pairs. Consequently, these approaches cannot fully exploit the comprehensive relationships between drugs and proteins.

With the rapid development of computing power and data mining, deep learning is widely accepted in the field of natural language processing and computer vision (Bharath Ramsundar et al. 2019, Hazra *et al.* 2021). Deep learning techniques have elevated traditional computational approaches due to the advantage of comprehensively extracting latent feature representations, rendering DTI prediction a research hotspot (Ru *et al.* 2021). Deep learning methods for inferring DTI are divided into two main types. One type is designed to manage a sequential input representation that transforms all feature information into a vector. For instance, DeepDTA (Öztürk *et al.* 2018) utilizes the Simplified Molecular Input Line Entry System (SMILES) of ligands and amino acid sequences of proteins to decide the binding affinity of drug–target pairs through convolutional neural networks (CNNs). Similarly, molecular transformer-based models (Shin *et al.* 2019, Huang *et al.* 2021) are introduced to determine the high-dimensional structure of a molecular from SMILES string and character-embedding sequence through the self-attention mechanism. However, these methods cannot model the potential relationships of compounds since the atom’s positional distribution and relative atoms are fixed, thereby limiting the prediction performance. Thus, a more flexible input representation of pairwise drug–target is required, another type of deep learning model, namely graph-based neural networks is frequently discussed. The application of graph descriptions in DTI prediction involves the treatment of atoms as nodes and chemical bonds as corresponding edges. Typically, GraphDTA (Nguyen *et al.* 2021) and IGT (Liu *et al.* 2022) are the outstanding identifiers for predicting DTI based on the graph representations of ligands and target receptors. Regrettably, most graph neural network (GNN)-based methods exhibit poor performance in extracting the spatial information of proteins through amino acid sequence representations, which is a staple factor in DTI determination. Proteins are composed of abundant atoms that require a large-scale sparse (3D) matrix to receive the entire constrained spatial structure. Hence, it is difficult to obtain an accurate protein high-resolution 3D structure. An alternative strategy was developed to handle the above-mentioned problem. In this strategy, the target proteins are expressed as a contact/distance map, which transforms the interaction among proteins into a matrix (Jiang *et al.* 2020, Zheng *et al.* 2020). However, this contact/distance map is based on a heuristic approach. Therefore, it provides only an abstract outcome of the true structure of proteins, which is generally distinguished from X-ray crystallography or nucleic magnetic resonance (NMR) spectroscopy (Tradigo 2013).

Several previously reported prediction models convert multi-scale neighboring topologies and diverse connections (i.e. interactions, associations, and similarities) among biological entities as feature representations to predict DTI. For example, GCDTI was proposed (Xuan *et al.* 2022a) to capture and fuse the neighboring topologies and diverse connections information based on three-way GCNs and CNN with multi-level attention. Similarly, Xuan *et al.* (2022b) used graph convolutional and variational autoencoders to encode multiple pairwise representations between a drug and its targets, such as attention-enhanced topology, attribution, and distribution. Unfortunately, the precision of these models in DTI prediction is relatively low because they ignored the molecular and chemical features of drug-protein pairs. Furthermore, existing deep learning methods always simply construct the interaction of drugs toward related targets by concatenating feature representations that cannot sufficiently express the interactions. Due to space constraints, we included more relevant studies in the Supplementary Materials.

Considering the above information, in this study, an end-to-end attention-derived method SAGDTI are introduced. SAGDTI accepts three input representations from multiple molecular and biological data sources, including the SMILES strings of drugs with relative atom distance, the graph embedding of proteins that contain spatial information of binding sites, and graph-based interaction information from biological heterogeneous network. First, we introduced a molecular transformer module based on multi-head self-attention (MSA) to extract the feature embedding of SMILES sequences and protein graphs. The feature representations of each drug–target pair were subsequently transformed into an interaction pairing map and we fed it into CNNs to represent the molecular attributes at the atom-level. Secondly, graph attention networks (GAT) are constructed to capture the interaction information that covers the multi-scale topologies and diverse connections in the form of a heterogeneous network (matrix) via graph-based attention. Finally, we use convolutional-pooling (C-P) networks to allocate the important weights of two obtained attribute representations and fully connected neural networks (FNNs) to predict the interaction score for drug–target pairs.

The main contributions of our study are summarized below.

- To accurately capture the unique molecular information of both compounds and receptors, a molecular transformer module is designed to improve the relative element information among atoms in drug compounds and 3D structural features of target proteins’ binding pockets.
- Our proposed model is the first DTI prediction method to transform the input representation of molecular drugs and targets into totally different forms. Moreover, it aggregates the attributes of interaction information from biological entities based on graph attention networks.
- Owing to its self-attention, the proposed method is attention-derived, offering high interpretability when aggregating the attribute representations from neither molecular nor biological feature information due to its self-attention.
- To our best cognition, our experimental results are state-of-the-art (SoTA) in feature representation learning models for DTI prediction, as confirmed using three benchmark datasets.

## 2 Methods

**Figure 1.**
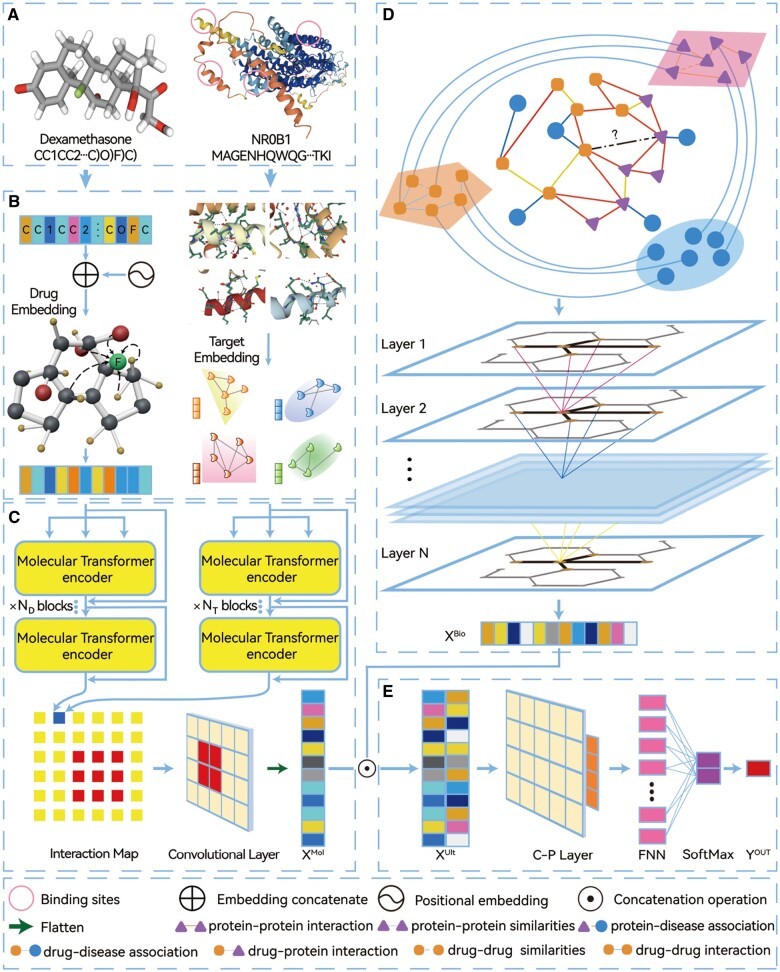
The framework of our proposed model. (A) Open-source data for drug SMILES strings and 3D structures of proteins. (B) Molecules input embedding module that identifies the noncovalent intermolecular attributes of drugs and proteins, and subsequently represents the features in the forms of sequences and graphs. (C) Molecular transformer module, in which we construct sequential transformer encoders to learn the unique feature representations of drugs SMILES and the binding sites of proteins, transform them into an interaction pairing map, and then feed them into a convolutional layer to extract molecular attributes. (D) Biological interaction information aggregating module that models the multi-scale topologies and diverse connections; the biological attributes are represented using a graph attention network. (E) Potential DTI classification module, where we predict unknown interactions in a drug–target pair.

In this study, we regarded the problem of predicting DTI as a binary classification issue. We model an objective function F(·) with multiple information representations for drugs and targets to forecast the presence or absence of DTI. The visualized framework of SAGDTI is shown in Fig. 1. Our model consists of four main parts: molecules input embedding; molecular transformer module; biological interaction information aggregating module and potential DTI classification module. Specifically, in molecule input embedding (Fig. 1B), we transformed the unique molecular information of drugs and proteins into different input forms. The SMILES strings of small compounds were embedded as sequential vectors that cover long-distance relative position information among atoms using the strategy introduced in (Zeng *et al.* 2021). Inspired by the graph input representation of proteins (Yazdani-Jahromi et al. 2022), the protein pockets with binding sites that interact with compounds were embedded as 3D graphs. Feature representations of drugs and proteins were subsequently fused to achieve the latent drug–target complex attributes that include their unique characteristics through the molecular transformer (Fig. 1C). Next, we modeled and aggregated the biological attributes of interaction information between drugs and targets by using GATs (Fig. 1D). Finally, the two obtained attribute representations were incorporated and then fed into C-Ps and FNNs for DTI prediction (Fig. 1E).

### 2.1 Molecules input embedding

#### 2.1.1 Drug SMILES strings

Small molecular drugs are expressed by the SMILES strings. Motivated by previous research (Zeng *et al.* 2021, Li *et al.* 2022), the SMILES strings were converted to continuous numerical vectors representing atoms and chemical information ({C,C,l,…,F,),C}→{42,42,25,…,11,31,42}). For a given drug: where *a_i_* is the *i*th atom and $N^{*}$ is a flexible sequence length that rests with atom numbers in a compound. In this study, we restrict the maximal SMILES sequence length as a hyperparameter $\ell_{d}$. According to the traditional transformer model (Vaswani *et al.* 2017), the initial input is an embedding vector composed of a tokenized symbol and position signal, which can be formulated as: where $E_{T}^{D}\in R^{\ell_{d}\times \varpi_{d}}$ and $E_{P}^{D}\in R^{\ell_{d}\times \varpi_{d}}$ represent the token embedding and position embedding, respectively, and $\varpi_{d}$ is the hidden feature dimension of atom *a_i_*. and *X^D^* denotes the embedding vector of the atom sequence. To model the relative distance between atoms, we describe the unique molecular characteristics using *m* kinds of relative correlations between atoms. Inspired by (Shaw *et al.* 2018), the input embedding of drugs with spatial information *D^In^* can be mathematically expressed as: where $W^{Q},W^{K},W^{V}\in R^{\varpi_{d}\times \varpi_{d}}$ denote parameter matrices of query, key and value in attention layer. $A^{R}\in R^{\ell_{d}\times \ell_{d}}$ indicates the relative-aware relationship matrix between atoms and $X_{i}^{D}$ is the *i*th atom embedding in *X^D^*. $W^{R}\in R^{m\times \varpi_{d}}$ is the learnable parameters composed of *m* kinds of relative relationship between atoms, and the virtual “nodes” (e.g. @, =, [, etc.) in *W^R^* are selected to be zero. Thus, when we model the relative distance between atoms, the distance between special characters in SMILES strings is 0.


(1) $$D=\{a_{1}^{d},a_{2}^{d},\ldots ,a_{i}^{d},\ldots a_{N^{*}}^{d}\}$$

(2) $$X^{D}=E_{T}^{D}+E_{P}^{D}$$

(3) $$D^{In}=\mathrm{SoftMax}(\frac{(X^{D}W^{Q}){\left(X^{D}W^{K}\right)}^{T}+A^{R}}{\sqrt{\varpi_{d}}})(X^{D}W^{V})$$

(4) $$A_{ij}^{R}=(X_{i}^{D}W^{Q})(W_{\min(k,|i-j|)}^{R})$$

#### 2.1.2 Protein graphs

We utilized the spatial structure of large-scale proteins that are collected from the Protein Data Bank (PDB) (Berman *et al.* 2000). This is a worldwide dataset that provides experimental evidence for proteins (e.g. through X-ray diffraction, cryo-electron microscopy, NRM). We sought to capture the binding sites of protein pockets from 3D structures. For this purpose, we adopted the algorithm introduced by (Saberi Fathi and Tuszynski 2014) to transform the bounding box coordinates of each protein’s binding site into a set of peptide fragments. For a given protein: where $b_{j}^{p}\in R^{\ell_{p}\times \upsilon }$ is the *j*th binding site in protein, $\ell_{p}$ and υ are the maximum number of binding sites and the maximum number of atoms in binding sites, respectively. $M^{*}$ denotes the varied number of protein binding sites. Since the binding sites of a protein were recognized, we drew individual graphs to represent each atom *a^p^* (node) and relationships between atoms (edges, denoted by *e^p^*) for each binding site. For each atom *a^p^*, we created a feature vector with a size of *k* (see Supplementary Table S1 for details), which comprised atom symbols, degree, electrical charge, hydrogen, etc. Thus, each binding site graph’s node matrix is denoted as $X^{P}\in R^{\upsilon \times k}$. Moreover, we use a simple linear transformation to encode $P^{In}=W^{P}{\left(X^{P}\right)}^{T}$, leading to a real-valued dense matrix $P^{In}\in R^{\upsilon \times D_{p}}$ as the input graph embedding. *D_p_* is the high-level feature dimension.


(5) $$P=\{b_{1}^{p},b_{2}^{p},\ldots ,b_{j}^{p},\ldots b_{M^{*}}^{p}\}$$

### 2.2 Molecular transformer module

In recent years, many attention-based algorithms have been developed to address the identification of drug-related targets, such as transformer (Vaswani *et al.* 2017) and BERT (Kang *et al.* 2022). Inspired by previous studies (Shin *et al.* 2019, Huang *et al.* 2021), we introduce a molecular transformer module to extract the feature representations of drugs and proteins, which cover their unique molecular characteristics.

Specifically, for the drug embedding representation *D^In^*, we fed it into a MSA layer to acquire the attention scores of each atom in the drug. We subsequently threw the output of MSA into a FNN layer, and both MSA and FNN layers are combined with residual operation (He *et al.* 2015) and layer normalization. The physical structure of the molecular transformer encoder is shown in Supplementary Fig. S1. Mathematically, the drug feature representation can be formulated as: where $Q\in R^{p\times e_{d}},\;K\in R^{p\times e_{d}}$ and $Q\in R^{q\times e_{v}}$ describe the a query and key-value pairs by attention function Att(). *head_i_* and *W_o_* are the *i*th head and learnable parameter matrix in MSA layer, respectively. *X^MSA^* is the output feature of MSA layer. Each molecular transformer encoder *MTs* was stacked using the residual operation to enhance molecular feature extraction, which is expressed as: where $D_{R}^{L-1}$ is the input of $L_{th}$ molecular transformer encoder, when $L-1=0,\;D_{R}^{L-1}$ equals to *D^In^*. Similarly, we model the latent feature representation of proteins $P_{R}^{L}$ from molecular graph embedding *P^In^* based on the transformer encoders.


(6) $$\mathrm{MTs}(D^{In})=\mathrm{FNN}(X^{\mathrm{MSA}}+D^{In})$$

(7) $$X^{\mathrm{MSA}}=\mathrm{Concate}(\mathrm{hea}d_{1},\mathrm{hea}d_{2},\ldots ,\mathrm{hea}d_{m})W_{o}$$

(8) $$\mathrm{hea}d_{i}=\mathrm{Att}(Q,K,V)$$

(9) $$\mathrm{Att}(Q,K,V)=\mathrm{SoftMax}(\frac{QK^{T}}{\sqrt{e_{d}}})V$$

(10) $$D_{R}^{L}=\mathrm{MTs}(D_{R}^{L-1})+D_{R}^{L-1}$$

To further learn the molecular attributes of drug-protein pairs at the atom-level, the two obtained feature representations for a given drug and its binding sites were converted into an interaction pairing map. Specifically, for each atom $a_{i}^{d}$ in a given drug and atom $a_{j}^{p}$ in a target protein, we constructed pairwise interactions via the following formula: where *IM* is an interaction pairing map and Λ(·) denotes the operation of the scalar product. Scalar products provide an interpretable approach to integrating the intensity of interaction between individual drug-protein pairs at the atom level. In *IM*, when a value is close to 1, the atoms of the corresponding pairwise small molecules are certainly binding with each other, otherwise, the value is 0. Thus, by utilizing this map, we explicitly determined the atoms of drug-protein pairs that contributed to the interaction result.


(11) $$IM=\Lambda (D_{R}^{L},P_{R}^{L})$$

A CNN layer was used to extract the adjacent information of neighboring atoms for DTI in *IM*. Let $W_{\mathrm{Conv}}\in R^{n_{w}\times n_{l}}$ be the convolutional filter, where *n_w_* and *n_l_* correspond to the width and length of the filter, respectively. There are *n_f_* channels to describe the molecular attributes of a drug and its binding protein in the CNN layer. The zero-padding technique is used to serve the marginal information of the atom binding features. Thus, the molecular attribute representations *X^Mol^* between drugs and targets are obtained via flattening operation.


(12) $$X^{\mathrm{Mol}}=\mathrm{Flatten}(\mathrm{Conv}(W_{\mathrm{Conv}}IM,n_{f}))$$

### 2.3 Biological interaction information aggregating module

In this module, we introduced GATs to model the multi-scale neighboring topologies and diverse connections from biological interaction information for improving the performance of DTI prediction. Generally, existing DTI prediction models only consider the noncovalent intermolecular interactions among SMILES strings and amino acid sequences. When some unexpected noise occurs, the accuracy may reduce significantly by the unavailability of biological feature information of drug-protein pairs. Therefore, we incorporated the biological characteristics of drug-protein pairs to increase the stability and robustness of the model. Numerous studies have demonstrated that the biological interaction information of drugs and targeted proteins plays an important role in predicting DTI (Peng *et al.* 2021, Zhao *et al.* 2021). The schematic of the GAT mechanism is shown in Supplementary Fig. S2.

Specifically, we first constructed a heterogeneous network, which contained all interaction information of multi-scale topologies and diverse correlations at the node-level. We converted the heterogeneous network into an undirected graph G=(V,E) where *V* represents different nodes and *E* represents the different edges between nodes. Afterward, the GAT layer was used to extract the input representation **h** of each node. where $\vec{h_{i}}$ and $K^{*}$ are the *i*th node and the number of adjacent nodes, and *Z* denotes the feature dimension. For each node $\vec{h_{i}}$, we used masked attention to calculate the attention coefficients, which means we only focus on the node $\vec{h_{i}}$ and its first-order neighboring node $\vec{h_{j}}\in N_{i}$. where *e_ij_* and *W* denote attention coefficients and the weight matrix of node feature transformation, respectively. Intuitively, the weights in GAT layer are shared, which greatly improve the efficiency in computation. The symbol ∪ denotes the concatenation operation of the two node representations, and $\alpha \in R^{2Z}$ is a weight vector. β(·) is the *LeakyReLU* activation function with a negative input slope of 0.2. The value of *e_ij_* reveals the importance of $\vec{h_{j}}$ to $\vec{h_{i}}$. To better allocate the weight and calculate the correlation strength between the central node $\vec{h_{i}}$ and all its neighbors, the attention coefficients are then normalized by utilizing a *SoftMax* function.


(13) $$h=\{\vec{h_{1}},\vec{h_{2}},\ldots ,\vec{h_{i}},\ldots ,\overset{–\to }{h_{K^{*}}}\},\vec{h_{i}}\in R^{Z}$$

(14) $$e_{ij}=\beta (\alpha^{T}[W\vec{h_{i}}\cup W\vec{h_{j}}])$$

(15) $$a_{ij}=\mathrm{SoftMa}x_{j}(e_{ij})$$

We sought to represent the interaction features more comprehensively between nodes. For this purpose, we also adopted the MSA strategy when normalizing the attention coefficients by using a nonlinear activation function. The output feature can be formulated as: where *k* means the *k_th_* head of attention coefficients $a_{ij}^{k}$.


(16) $$\vec{h\prime_{i}}=\overset{\mathrm{Head}}{\underset{k=1}{\cup }}\beta (\sum_{j\in N_{i}}a_{ij}^{k}W^{k}\vec{h_{j}})$$

After extracting the complex feature representation of interaction information between a drug and its target through consecutive GAT layers, we then utilized a global max pooling to output the biological attribute representation vector. The output representation *X^Bio^* is expressed as:


(17) $$X^{\mathrm{Bio}}=\mathrm{MaxPoolng}(\vec{h\prime_{i}})$$

### 2.4 DTI classification module

Most existing methods only simply concatenate the learned feature representations or include them into several FNN layers. These cannot fully learn the real-world interaction knowledge between a drug and its binding target. Inspired by Zeng *et al.* (2021) in modeling the DTI feature, C-P and FNN layers are applied to map the extracted features into a final classification output. Firstly, the molecular attribute representations *X^Mol^* and the biological attribute representations *X^Bio^* were spliced left and right to fuse the ultimate feature representation *X^Ult^*.


(18) $$X^{\mathrm{Ult}}=[X^{\mathrm{Mol}};X^{\mathrm{Bio}}]$$

Secondly, the fused attribute representations *X^Ult^* of a drug–target pair was fed into C-P and FNN layers to learn the whole DTI features, and a *Sigmoid* function was used to output the final interaction score. where $W\prime_{\mathrm{Conv}}$ and $n\prime_{f}$ denote the convolutional kernel and corresponding channels, respectively, S represents the *Sigmoid* function, in which $S(x)=\frac{1}{1+\text{exp}(-x)}$. We applied the Adam algorithm to optimize the objective and utilize the binary cross-entropy (BCE) loss function to minimize the difference between the ground truth and the predicted probability, which can be calculated as: where *λ* is the number of training samples and $Y_{i}^{*}$ represents the real label of a drug–target pair. When the value of *Loss* is close to 0, it indicates the SAGDTI model can predict the DTI with high accuracy.


(19) $$Y^{\mathrm{Out}}=S(\mathrm{FNN}(\mathrm{Pool}(\mathrm{Conv}(W\prime_{\mathrm{Conv}}X^{\mathrm{Ult}},n\prime_{f}))))$$

(20) $$\mathrm{Loss}=-\frac{1}{\lambda }\sum_{i=1}^{\lambda }Y_{i}^{*}\cdot \log(Y_{i}^{\mathrm{Out}})+(1-Y_{i}^{*})\cdot \log(1-Y_{i}^{\mathrm{Out}})$$

## 3 Results

### 3.1 Benchmark datasets

In this study, we evaluated the merit of our proposed model using three benchmark datasets, BindingDB dataset (Gilson *et al.* 2016), Kinase dataset Davis (Davis *et al.* 2013), and Kinase Inhibitor BioActivity (KIBA) (Tang *et al.* 2014), which are widely utilized in previous studies (Cheng *et al.* 2022, Li *et al.* 2022). The SMILES strings of molecular drugs are collected from the PubChem database (Kim *et al.* 2021) based on their PubChem IDs, the 3D structure information of proteins is derived from the PDB database (Berman *et al.* 2000), and the interaction information of drug–target pairs is obtained according to prior work (Zhao *et al.* 2021).In this article, when the structure information of a protein is not available in the PDB database, we will remove it to make sure the model can fully learn the interaction features of drug-protein pairs. Table 1 summarizes the statistical information for these datasets. These datasets are processed to filter out the invalid DTIs for data processing (see Supplementary Materials).

**Table 1.** Statistics of the three benchmark datasets: BindingDB dataset, Davis and KIBA.

Datasets	Metric	Target	Drug	Interaction
	*K_D_*	587	6704	31 239
BindingDB	*K_I_*	1225	126 122	249 598
	*IC*_50_	2582	407 462	653 344
	*EC*_50_	699	75 142	105 364
Davis	*K_D_*	442	68	30 056
KIBA	KIBA score	229	2111	118 254

### 3.2 Evaluation criteria

To estimate the utility of our DTI prediction model, we used five metrics in the experiments in total, which are area under the receiver operating characteristics curve (AUROC), area under the precision–recall curve (AUPR), Matthews correlation coefficient (MCC) (He *et al.* 2022), *F*_1_-score, and balanced accuracy (B.Acc, composed of sensitivity and specificity). The mathematical formula of the above five metrics is displayed in the Supplementary Materials.

### 3.3 Experiments setup

To comprehensively evaluate the performance of DTI prediction, we adopted three experimental settings, i.e. a new-target setting, a new-drug setting, and a pairwise setting. In the new-target setting, targets are randomly split into five identical subsets, four of those are regarded as inference targets, while the remaining subset is treated as test targets. In the training process, the SAGDTI model learns the inference data, which contains the inference targets and all the drugs. Subsequently, the trained model is used to suggest the pairwise interactions between test targets and all the drugs. In this regard, the new-drug setting is analogous to the new-target setting, and we arbitrarily divide the drugs into five groups. The SAGDTI model is trained and applied to predict the interaction between new drugs and all the targets. In the pairwise setting, the known DTI is like drugs or targets, which are grouped as five equal folds.

Similar to the Co-VAE, we clipped the dataset as training data and testing data at a 5:1 ratio. The training data were used as a training set and a validation set to identify the optimal parameters through a 5-fold cross-validation strategy. For each setting, we applied the trained SAGDTI model on the test set and repeated this process five times to obtain the final mean and standard deviation results. We adopted identical settings for impartial comparison when comparing our proposed model with existing methods. To perform the SAGDTI model, several hyperparameters are shown in Table 2 (See Supplementary Materials for training details).

**Table 2.** Statistics of the three benchmark datasets: BindingDB dataset, Davis and KIBA.

Parameters	Range
Max length (drug)	150
Max length (target)	1200
Attention head in MTs (drug)	[4–12]
Attention head in MTs (target)	[4–12]
Attention head in GAT	[8–12]
Filter size	3*3
Number of filters	[32, 64, 96]
Kernel size of the pooling layer	2*2
Dropout	[0.1–0.6]
Optimizer	Adam
Learning rate	[0.1, 0.01, 0.001, 0.0001]
Epoch	[30, 50, 100]
Batch size	[32, 64, 128, 256]

### 3.4 Comparison with SoTA methods

The performance of the proposed SAGDTI for DTI prediction was compared with several SoTA methods, including DDR (Olayan *et al.* 2018), DeepDTA (Öztürk *et al.* 2018), GraphDTA (Nguyen *et al.* 2021), IGT (Liu *et al.* 2022), Moltrans (Huang *et al.* 2021), AttentionSiteDTI (Yazdani-Jahromi et al. 2022), MATTDTI (Zeng *et al.* 2021), and Co-VAE (Li *et al.* 2022). For a fair comparison, we used the same experimental settings on all the models and the original hyperparameter reported in the corresponding publications. Details of the compared models can be found in Supplementary Materials.

**Figure 2.**
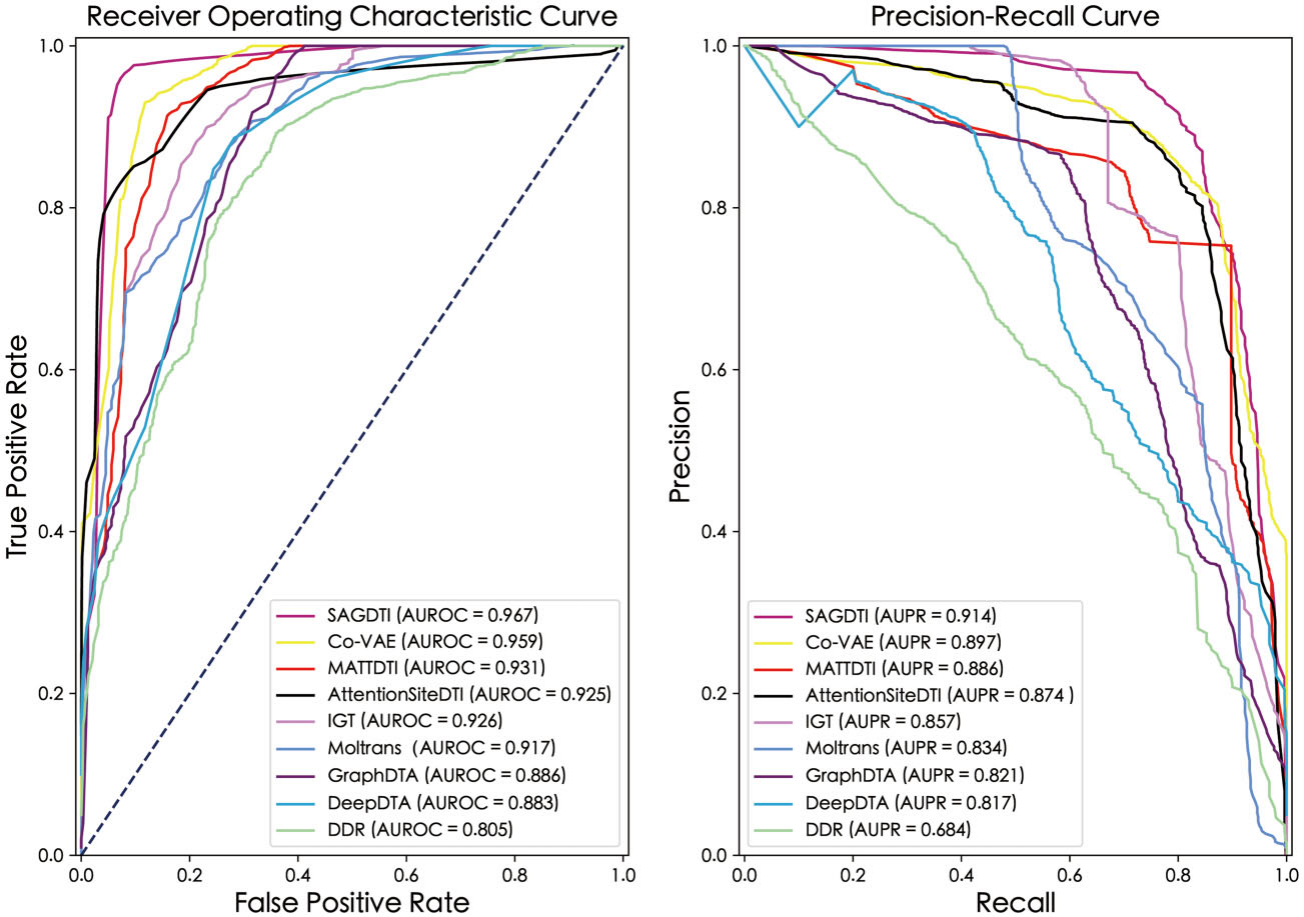
Comparison of the SAGDTI with eight SoTA models in AUROC and AUPR metrics. Left: ROC curves of different DTI models. Right: PR curves of different DTI models.

We first adopted the BindingDB dataset to evaluate the SAGDTI model by drawing the receiver operating characteristics curve and precision–recall curve, which record the highest performance in the experimental results. As plotted in Fig. 2, our proposed model SAGDTI achieved the best AUROC and AUPR values compared with other advanced methods. Specifically, SAGDTI obtained the best performance with 0.967 (AUROC) and 0.917 (AUPR), which is 0.008 and 0.017 superior to in the second-best model, respectively. The results roughly show that SAGDTI specializes in inferring the interaction of pairwise drug–target. In the BindingDB dataset, the training set and test set were extremely unbalanced, indicating that the AUPR metric is more credible for expressing the prediction performance. The results for three experimental settings are presented in Table 3. According to the AUROC performance, SAGDTI achieved the best values and was 1.8%, 0.97%, and 1.9% better than other prediction models in terms of three settings (i.e. new-target setting, new-drug setting and pairwise setting). Furthermore, we acquired the average performance of the five metrics among the three settings based on the BindingDB dataset (Table 4). It demonstrated that SAGDTI is competent enough in DTI prediction, as reflected by the average metric values obtained under three experimental settings: AUROC (0.967), AUPR (0.914), MCC (0.649), F1-Score (0.861), and B.Acc (0.901).

**Table 3.** Comparison of SAGDTI with other eight SoTA methods in AUROC and AUPR metrics under three different experimental conditions, using three benchmark datasets.^a^

Datasets	Methods	New-target		New-drug		Pairwise	
		AUROC (std)	AUPR (std)	AUROC (std)	AUPR (std)	AUROC (std)	AUPR (std)
BindingDB	DDR	0.834 (0.033)	0.747 (0.051)	0.827 (0.037)	0.725 (0.062)	0.823 (0.031)	0.719 (0.054)
	DeepDTA	0.868 (0.007)	0.823 (0.011)	0.864 (0.006)	0.776 (0.0011)	0.842 (0.006)	0.811 (0.009)
	GraphDTA	0.875 (0.008)	0.835 (0.0010)	0.852 (0.009)	0.783 (0.013)	0.851 (0.006)	0.829 (0.009)
	Moltrans	0.893 (0.011)	0.842 (0.015)	0.878 (0.012)	0.812 (0.013)	0.858 (0.009)	0.847 (0.011)
	IGT	0.912 (0.007)	0.855 (0.011)	0.891 (0.009)	0.833 (0.015)	0.887 (0.006)	0.852 (0.009)
	AttentionSiteDTI	0.937 (0.005)	0.883 (0.009)	0.912 (0.006)	0.853 (0.011)	0.908 (0.005)	0.871 (0.007)
	MATTDTI	0.928 (0.004)	0.875 (0.008)	0.907 (0.005)	0.864 (0.007)	0.906 (0.003)	0.869 (0.005)
	Co-VAE	0.955 (0.002)	0.897 (0.004)	0.921 (0.001)	**0.893 (0.002)**	0.914 (0.002)	0.884 (0.003)
	SAGDTI	**0.967 (0.001)**	**0.914 (0.002)**	**0.934 (0.002)**	0.886 (0.002)	**0.946 (0.001)**	**0.901 (0.001)**
Davis	DDR	0.784 (0.035)	0.451 (0.067)	0.763 (0.033)	0.407 (0.056)	0.739 (0.023)	0.411 (0.049)
	DeepDTA	0.799 (0.013)	0.526 (0.021)	0.773 (0.015)	0.491 (0.022)	0.752 (0.011)	0.485 (0.018)
	GraphDTA	0.816 (0.015)	0.547 (0.026)	0.802 (0.011)	0.514 (0.019)	0.783 (0.013)	0.501 (0.019)
	Moltrans	0.849 (0.016)	0.577 (0.022)	0.826 (0.014)	0.531 (0.021)	0.815 (0.012)	0.524 (0.17)
	IGT	0.873 (0.013)	0.593 (0.023)	0.849 (0.013)	0.546 (0.019)	0.839 (0.011)	0.537 (0.018)
	AttentionSiteDTI	0.902 (0.009)	0.601 (0.017)	0.872 (0.011)	0.578 (0.020)	0.865 (0.008)	0.561 (0.0.015)
	MATTDTI	0.917 (0.006)	0.625 (0.014)	0.905 (0.007)	0.612 (0.019)	0.873 (0.005)	0.594 (0.009)
	Co-VAE	0.922 (0.003)	0.644 (0.009)	0.911 (0.005)	**0.641 (0.009)**	0.886 (0.002)	**0.632 (0.003)**
	SAGDTI	**0.937 (0.002)**	**0.645 (0.005)**	**0.921 (0.003)**	0.636 (0.004)	**0.903 (0.001)**	0.627 (0.002)
KIBA	DDR	0.792 (0.024)	0.446 (0.041)	0.773 (0.027)	0.416 (0.040)	0.746 (0.019)	0.404 (0.035)
	DeepDTA	0.804 (0.007)	0.517 (0.015)	0.784 (0.008)	0.485 (0.017)	0.765 (0.008)	0.492 (0.015)
	GraphDTA	0.824 (0.009)	0.556 (0.014)	0.806 (0.007)	0.512 (0.013)	0.799 (0.006)	0.516 (0.011)
	Moltrans	0.856 (0.008)	0.583 (0.015)	0.831 (0.006)	0.558 (0.014)	0.827 (0.007)	0.567 (0.010)
	IGT	0.886 (0.008)	0.607 (0.013)	0.853 (0.007)	0.601 (0.015)	0.841 (0.007)	0.591 (0.013)
	AttentionSiteDTI	0.907 (0.006)	0.625 (0.012)	0.886 (0.007)	0.615 (0.013)	0.864 (0.006)	0.586 (0.009)
	MATTDTI	0.921 (0.005)	0.632 (0.009)	0.914 (0.005)	0.627 (0.010)	0.889 (0.004)	0.619 (0.007)
	Co-VAE	0.932 (0.002)	0.645 (0.003)	0.918 (0.001)	**0.651 (0.003)**	0.894 (0.001)	0.645 (0.002)
	SAGDTI	**0.945 (0.001)**	**0.657 (0.002)**	**0.927 (0.001)**	0.643 (0.002)	**0.911 (0.001)**	**0.645 (0.001)**

a Bold: best results.

**Table 4.** Performance evaluation for predicting DTI on the BindingDB dataset using the average values of five metrics.^a^

	AUROC	AUPR	MCC	F_1_score	B.Acc
DDR	0.805 (0.037)	0.684 (0.057)	0.425 (0.071)	0.529 (0.043)	0.758 (0.035)
DeepDTA	0.883 (0.009)	0.817 (0.011)	0.597 (0.046)	0.654 (0.037)	0.811 (0.026)
GraphDTA	0.886 (0.007)	0.821 (0.010)	0.584 (0.041)	0.676 (0.034)	0.819 (0.027)
Moltrans	0.917 (0.007)	0.834 (0.013)	0.605 (0.031)	0.669 (0.035)	0.823 (0.031)
IGT	0.926 (0.008)	0.857 (0.011)	0.631 (0.035)	0.817 (0.031)	0.842 (0.028)
AttentionSiteDTI	0.925 (0.006)	0.874 (0.009)	0.627 (0.029)	0.840 (0.027)	0.857 (0.019)
MATTDTI	0.931 (0.005)	0.886 (0.004)	0.644 (0.021)	0.833 (0.021)	0.849 (0.016)
Co-VAE	0.959 (0.002)	0.897 (0.003)	**0.653 (0.012)**	0.856 (0.019)	0.871 (0.011)
SAGDTI	**0.967 (0.002)**	**0.914 (0.002)**	0.649 (0.013)	**0.861 (0.016)**	**0.901 (0.011)**

a Bold: best results.

Existing prediction models (e.g. Co-VAE, MATTDTI, and AttentionSiteDTI) also exhibited excellent performance. The Co-VAE model introduced a novel co-regularized variational autoencoder framework with a strong generative capability. Co-VAE can retain all the learned DTI features by generating the SMILES strings and amino acid sequences to model the original drug or target features. Thus, Co-VAE demonstrated an excellent performance in the identification of DTIs. However, the Co-VAE model ignored the unique molecular information and simply fused the features of drugs and targets through concatenation. MATTDTI considered the long-distance relative relationships among atoms in drugs, and used multi-head attention to compute the DTI similarities. The AttentionSiteDTI method took advantage of the 3D structure information based on the binding sites of proteins and utilized an attention-based module to represent the output vector. All these models captured the DTI features without fully exploiting the unique molecular feature attributes. We speculate that SAGDTI outperformed other SoTA models because it included the whole unique characteristics of both drugs and targets, as well as aggregated the neighboring topologies and diverse connections. In summary, we intuitively compared the prediction capability of the eight models using the curve plots and different experimental settings. Our SAGDTI exhibited the highest performance overall.

We further used the Davis and KIBA datasets to further evaluate the prediction performance of our SAGDTI model. In the Davis dataset, our model almost exhibited the best performance in each experimental setting. In the new-drug setting, it ranked second only to the Co-VAE model in terms of the AUPR value. As shown in Table 3, SAGDTI obtained 1.6%, 1.1%, and 1.9% enhancement in the AUROC metric under the new-target, new-drug, and pairwise settings, respectively. In terms of AUPR values, the performance of all methods was markedly decreased due to the obvious shortage of DTI training samples in the Davis dataset. However, our proposed model is still SoTA, like the Co-VAE model performs in the new-drug and pairwise settings. We also noted excellent performance in the KIBA dataset with AUROC values of 0.945, 0.927, and 0.911, and AUPR values of 0.657, 0.643, and 0.645 for the corresponding settings, respectively.

Generally, the high accuracy of random split settings is known to be overestimated, not a model’s real-world prediction performance. When there is little information about the interaction between a protein and a drug, the learning performance of most models drops greatly. Thus, we adopted a type of nonoverlapped sampling splitting strategy (i.e. cold start splitting) for better evaluation. Inspired by (Atas Guvenilir and Doğan 2023), we used the modified Davis (mDavis) dataset to make sure the model can learn general principles from data rather than memorizing it. We randomly select 10% or 15% DTI pairs for test samples and remove all of their associated drugs and proteins from the training samples. The results in Table 5 indicate that all methods have a great performance decline in cold start splitting. However, SAGDTI still achieves the best performance against other SoTA baselines, and, interestingly, the enhancement is even more obvious. According to the overall experimental results we obtained, it can be easily concluded that SAGDTI outperforms existing SoTA models in the task of DTI prediction. We think that our SAGDTI model can be significantly improved due to the full consideration of the unique noncovalent intermolecular attributes at the atom-level and biological interaction attributes at the node-level between drugs and targets.

**Table 5.** The results of cold start splitting on mDavis dataset.^a^

Methods	Remove 10%		Remove 15%	
	AUROC	AUPR	AURPC	AUPR
DeepDTA	0.780	0.501	0.728	0.453
GraphDTA	0.778	0.526	0.732	0.511
Moltrans	0.805	0.564	0.773	0.547
MATTDTI	0.809	0.584	0.785	0.564
Co-VAE	0.816	0.605	0.796	0.585
SAGDTI	**0.863**	**0.617**	**0.843**	**0.604**

a Bold: best results.

### 3.5 Ablation study

To ensure the stability and robustness, we also conducted a comprehensive ablation study on contribution of different modules in SAGDTI. The AUROC and AUPR were applied to evaluate the effectiveness of five SAGDTI variants (see Supplementary Materials for detail descriptions). For example, the SAGDTI *II* means the biological interaction information aggregating module was removed from the proposed model. Through this step, we overlooked the attributes of topologies and diverse connections between drugs and targets, and considered only the molecular attributes. Consequently, the performance of SAGDTI (without GATs) has declined markedly (see Supplementary Tables S3 and S4). In our work, we introduce multi-sources input information for SAGDTI, and thus prior knowledge (e.g. the drug SMILES sequence, protein 3D structure, and multi-scale DTI information) may be produced. To avoid an over-optimistic evaluation of the proposed method, we removed the different input elements accordingly. The performance of SAGDTI with different input information is shown in Supplementary Table S5. Obviously, the performance of our proposed model exhibits a downward trend to some extent without adequate input source. However, SAGDTI still has a competitive performance in identifying the interaction between drugs and targets, though we neglect the protein 3D structure and multi-scale biological information simultaneously. Supplementary Table S5 indicates that comprehensively consider the molecular and biological information is beneficial for learning high-level feature representations of drugs and targets and for improving prediction performance. These results also demonstrated that our SAGDTI has a good generalization ability.

### 3.6 Case study

Since December 2019, the infection of the new coronavirus (SARS-CoV-2) has rapidly spread worldwide, leading to a pandemic (Kirtipal *et al.* 2020). Hence, there is an urgent need to identify effective drugs for patients infected with SARS-CoV-2. Thus, we enforced case studies on five SARS-CoV-2-related drugs (i.e. Remdesivir, Lopinavir, Budesonide, Dexamethasone and Aripiprazole) to further verify the capability of SAGDTI for determining possible DTI. Each drug interacts with at least 12 different targets. Since space limitation, the top 10 candidate proteins identified by SAGDTI for each drug were listed in Supplementary Table S6.

For brevity, we discuss below our findings for the drugs remdesivir and lopinavir. Remdesivir (a nucleoside analog) possesses antiviral activity with *EC*_50_ values of 74 nM against SARS-CoV and Middle East Respiratory Syndrome Coronavirus (MERS-CoV) in Hereditary angioedema (HAE) cells and 30 nM against murine hepatitis virus in delayed brain tumor cells (Olender *et al.* 2021). In animal and in vitro models, remdesivir has demonstrated activity against the viral pathogens of SARS and MERS, which are also coronaviruses and are structurally very similar to SARS-CoV-2 (Kokic *et al.* 2021). We ranked the top 10 targets that are verified to interact with the remdesivir. The top 2 targets, which are the Replicase polyprotein 1ab (Rep) and the RNA-directed RNA polymerase L (L), happen to be the genome sequences of SARS-CoV-2-related disease. Lopinavir is a protease inhibitor of the human immunodeficiency virus (HIV)-1 and HIV-2. It is combined with ritonavir for the treatment of HIV infection (Rebello *et al.* 2018). Through experiments, we identified a SARS-CoV-2-related target Pol polyprotein (Pol) that interacts with lopinavir. According to the results, our proposed prediction model can assist scientists in drug development and clinicians in real-life practice.

## 4 Discussion

We have demonstrated the advantageous performance of SAGDTI through the evaluations of the BindingDB, Davis and KIBA datasets under three experimental settings and the SARS-CoV-2 case study. The experimental results proved that our proposed SAGDTI model performs well in DTI prediction and may be useful in practical applications. However, there are still some problems that have not been explained clearly and some difficulties need to be solved urgently. Such as, how to obtain the accurate quantification of the binding affinity (interaction strength) when interaction happens, where is the docking position in 3D space and whether the docking postures matter to the DTIs in a real-life situation (Wei *et al.* 2022). In this article, we treat the DTI prediction problem as a binary classification, which simply suggests the absence or presence of interactions. Therefore, we intend to expand our research on the drug–target binding affinity and investigate the mechanisms through which the docking position and posture influence the results. We believe that our proposed model can perform well in practical prediction for predicting DTIs and improve the development of drug–target binding affinities in the future.

## 5 Conclusion

In this article, we proposed an end-to-end attention-derived model, termed SAGDTA. The aim of this model is to capture the unique molecular attributes of drugs as well as targets and aggregate the biological interaction information of drug–target pairs to predict the DTI. The proposed framework enables learning the SMILES sequence with the relative distance among atoms of a given drug and the 3D structural feature of proteins from their binding sites at the atom-level through the molecular transformer module. SAGDTI also allows the aggregation of neighboring topologies and diverse connections (i.e. interactions, associations, and similarities) at the node-level based on the GATs. Experimental results demonstrated that, in most cases, our proposed SAGDTI model could predict DTI with superior performance than other advanced models (e.g. Co-VAE, MATTDTI, and AttentionSiteDTI) under three experimental settings. Furthermore, we used the trained model to predict the interactions of five SARS-CoV-2-related drugs with potential targets to assist scientists. Our model displayed SoTA performance, was highly interpretable based on the self-attention mechanism and performed well in a real-life case study. The limitations and advantages of SAGDTI have been fully discussed. We believe that this work could contribute to the research of drug discovery as well as drug repurposing, and be a powerful tool in practical DTI prediction.

## Supplementary Material

vbad116_Supplementary_Data
