# Supplementary material for: SAGDTI: self-attention and graph neural network with multiple information representations for the prediction of drug–target interactions
Source: Bioinform Adv. 2023 Aug 26;3(1):vbad116. doi: 10.1093/bioadv/vbad116 (PMC10818136; doi:10.1093/bioadv/vbad116)
Supplement: vbad116_Supplementary_Data [file vbad116_supplementary_data.pdf]

Supplementary Information

SAGDTI: self-attention and graph neural network with multiple information representations for drug-target interaction prediction

Xiaokun Li[2, 3], Qiang Yang[2,3], Gongning Luo[1, *], Long Xu[2, 3], Weihe Dong[3, 4], Wei Wang[1], Suyu Dong[4], Kuanquan Wang[1, *], Ping Xuan[2,5] and Xin Gao[6]

[1]School of Computer Science and Technology, Harbin Institute of Technology, West Dazhi Street, 150001, Harbin, China.
[2]School of Computer Science and Technology, Heilongjiang University, Xuefu Road, 150080, Harbin, China.
[3]Postdoctoral Program of Heilongjiang Hengxun Technology Co., Ltd., Xuefu Road, 150090, Harbin, China.
[4]College of information and Computer Engineering, Northeast Forestry University, Hexing Road, 150040, Harbin, China.
[5]Department of Computer Science, School of Engineering, Shantou University, Daxue Road, 515063, Shantou, China.
[6]Computer, Electrical and Mathematical Sciences & Engineering Division, King Abdullah University of Science and Technology, 4700 KAUST, Thuwal 23955, Saudi Arabia.
*Corresponding authors:
Gongning Luo, Biocomputing Research Center, School of Computer Science and Technology, Harbin Institute of Technology, 150001, Harbin, China; E-mail: luogongning@hit.edu.cn；
Kuanquan Wang, Biocomputing Research Center, School of Computer Science and Technology, Harbin Institute of Technology, 150001, Harbin, China; E-mail: wangkq@hit.edu.cn.

Related Work

In the present investigation, deep learning-based methods were evolved to overcome the challenge of modeling DTI prediction. These deep learning methods differ considerably in their architecture and the manner in which they represent the input data. In many of them [1]-[5], the task of DTI identification is considered a binary classification problem.

As our article mentioned, information on the 3D structure of proteins can be easily and effectively captured by CNN, however, long-distance relative atoms for small molecules of compounds are neglected. Recent studies have proposed solutions to this problem. For example, Zeng et al. [6] developed an end-to-end model, named MATTDTI, which contains a relation-ware self-attention block to collect all element information among atoms of drugs. AtomNet [7], the first sequence-based method, utilizes 3D CNN to extract arbitrary molecular features for the modeling of the binding site between the ligand and its target, which requires the spatial position of each atom. Li et al. [8] proposed co-regularized variational autoencoders that regard drug structures and target sequences as input representations to generate the binding affinities. This method outperformed the DeepAffiniy [9] and DeepDTA in terms of maximizing the lower bound triplet likelihood of drugs, targets, and their affinities.

With the critical breakthrough in deploying GCN methods for DTI prediction [10]-[14] numerous graph-based input representations have been adopted to express the high-quality structural position of proteins. AttentionSiteDTI [15] an attention-enhanced DTI prediction model, constructed a Graph Attention Embedding Module to model the binding sites of proteins and chemical information of small molecule drugs by forming input representations as bidirectional graphs. Torng et al. [16] proposed an unsupervised deep learning model, called Pocket Feature, to learn the features of 3D graph representations for protein binding sites as well as two-dimensional graph representations for drug SMILES strings. Besides, TriModel was proposed by Sameh et al. [17] suggesting the occurrence or absence of interactions based on biomedical knowledge graph embedding.

Data source

In this work, we applied three benchmark datasets to evaluate the performance of the end-to-end attention-derived deep learning model for drug-target interaction prediction, namely, the bindingDB dataset [18], Davis [19] and KIBA [20]. What's more, we also utilized the PubChem database [21], the Protein Data Bank database [22] and Luo et al. database [23] as tools to process the feature information of compounds and proteins.

BindingDB is an open-source database, which covers experimental-based assays to determine the binding strength between drugs and proteins. We used the universal subset of the dataset judged by the dissociation constant K_D that contains 31,239 DTI samples among 6,704 drugs and 587 proteins. We confirmed that the interaction between a compound and its protein existed if the corresponding K_D value was < 30 units. The Davis dataset comprised 68 ligands and 442 proteins for 30,056 DTI samples. It describes the binding affinities of the kinase protein cluster and the correlated inhibitors with their respective K_D values. The KIBA dataset uses a KIBA binding score, which is a statistical combination of kinase inhibitor bioactivities from the K_D, inhibition constant K_I, and half-maximal inhibitory concentration (IC_{50}), to identify the interactions between drugs and targets. We set the binarization threshold of KIBA scores as KIBA $\leq$ 3, i.e., if the score is ≤ 3, the DTI is equal to 1. The dataset originally comprised 467 targets and 52,498 drugs. According to He et al. [24], we filter it to involve 2,111 unique drugs and 229 unique targets, with a ratio of 24.4% on affinity quantity density.

PubChem (http://pubchem.ncbi.nlm.nih.gov) is a public repository for biological activity data of small molecules and RNAi reagents. The mission of PubChem is to deliver free and easy access to all deposited data, and to provide intuitive data analysis tools. The PubChem BioAssay database currently contains 500,000 descriptions of assay protocols, covering 5000 protein targets, 30,000 gene targets and providing over 130 million bioactivity outcomes. In this study, we use it to collect the SMILES strings and chemical feature information of drugs.

The Protein Data Bank (PDB; http://www.rcsb.org/pdb/) is the single worldwide archive of structural data of biological macromolecules. We use PDB to describe the 3D structure information of proteins, such as coordinates, covalent bond distances and angles.

Luo et al. database (https://github.com/luoyunan/DTINet) contains several types of biological entities (nodes), including 708 drugs, 1,512 proteins and 5,603 diseases, which can construct complex networks with seven edges, namely, drug-protein interactions, drug-drug interactions, drug-drug similarities, drug-disease interactions, protein-disease associations, protein-protein interactions and protein-protein similarities. We use it to extract the biological interactive information to elevate the ceiling boundary of SAGDTI's prediction performance.

Evaluation metrics

AUROC (area under receiver operating characteristics) is a widely used evaluation metric for classification tasks indicates the model's distinguishing ability between two classes. AUPC stands for the area under the precision-recall curve and is an effective metric to estimate the ability to detect positive samples from imbalanced datasets.

The Matthews correlation coefficient (MCC) takes into account TP, TN, FP and FN, which is generally regarded as a balanced measure that can be used even if the classes are of very different sizes. MCC is defined as:

$$MCC = \frac{TP \times TN - FP \times FN}{\sqrt{(TP + FP)(TP + FN)(TN + FP)(TN + FN)}}$$

F_1-Score is a statistical measure of the accuracy of binary classification models. It considers the precision and recall of the classification model at the same time. It is defined as follows:

$$F_1 - Score = 2 * \frac{Precision * Recall}{Precision + Recall}$$

The balanced accuracy (B.Acc) calculates the balanced accuracy, which avoids inflated performance estimated on imbalanced datasets. B.Acc is defined as the average between specificity and sensitivity at a certain threshold:

$$Sensitivity = \frac{TP}{TP + FN}, Specificity = \frac{TN}{FP + TN}$$

$$B.Acc = \frac{1}{2}(Specificity + \ Sensitivity)$$

Training process

In this work, we applied a grid search over a range of values to select the hyperparameters of our model. All hyperparameters were tuned to yield the best result for each dataset. In molecules input embedding model, the max length of drug SMILES strings and amino acid sequences are restricted to 100 and 1200, respectively. In molecular transformer module, the number of attention head is 8 for both drugs and targets, and the hidden layer size is 500. All the kernel size of the convolutional neural network are 3, and the number of kernels is 64. The output dimension of molecular transformer module is set to 128. In the biological interactive information aggregating module, the attention head is set to 6 and the output dimension is 128. Adam is selected as the optimizer and the batch size is set to 256. The number of different network layers for each dataset is summarized in Table S2.

The drug-target interaction prediction models are trained for 100 epochs, and we choose the checkpoints with the lowest validation errors for evaluation.

Baseline

In this paper, we compared SAGDTI with eight cutting-edge prediction methods. They are DDR [25], DeepDTI [1], GraphDTI [10], IGT [26], Moltrans [27], AttentionStieDTI [15], MATTDTI [6] and Co-VAE [8].

DDR is based on the use of a heterogeneous graph that contains known DTIs with multiple similarities between drugs and multiple similarities between target proteins. DDR applies non-linear similarity fusion method to combine different similarities.

DeepDTA employs three-layers CNNs as Protein Encoder and Compound Encoder to encode the protein sequences and the compound SMILES strings, respectively. Then, for the Interaction Estimator, the encoded protein and compound are concatenated to predict the affinity score.

GraphDTA regards each compound as a graph and attempts several GNNs, such as GIN, GAT, GCN and GAT-GCN, as the Compound Encoders to represent the compounds. In the meantime, GraphDTA regards each protein as a sequence and adopts CNNs as the Protein Encoder to encode the proteins. Then, the feature representations of drugs and proteins are combined and fed into two fully connected layers to estimate the interaction score.

IGT takes as input the 3D structure of the complex after docking, and first goes through a feature extraction module to obtain graph representations of the complex, protein, and small molecule, respectively. Then the three molecular graphs as well as the initialized features are fed into a message-passing module consisting of tandem repeated building blocks. In each building block, three Graph Transformers are designed to extract three kinds of interactive information respectively, i.e., intramolecular interactions of the ligand (the ligand graph), intramolecular interactions of the receptor (the receptor graph), and more importantly, the intermolecular interactions between the receptor and the ligand (complex graph).

Moltrans is a Molecular Interaction Transformer (MolTrans) to address two main DPI prediction limitations. It used knowledge inspired sub-structural pattern mining algorithm and interaction modeling module for more accurate and interpretable DTI prediction. Moltrans applied an augmented transformer encoder to better extract and capture the semantic relations among substructures extracted from massive unlabeled biomedical data.

AttentionSiteDTI is inspired by sentence classification models in the field of Natural Language Processing, where the drug-target complex is treated as a sentence with relational meaning between its biochemical entities a.k.a. protein pockets and drug molecules. It enables interpretability by identifying the protein binding sites that contribute the most toward the drug-target interaction.

MATTDTI is an end-to-end model with multiple attention blocks to predict the binding affinity scores of drug-target pairs. It offers the ability to encode the correlations between atoms by a relation-aware self-attention block and model the interaction of drug representations and target representations by the multi-head attention block.

Co-VAE is novel co-regularized variational autoencoders (Co-VAE) to identify drug-target binding affinity based on drug structures and target sequences. It can maximize the lower bound of the joint likelihood of drug, protein and their affinity based on KL Divergence for different data distribution, mathematically.

$$\mathcal{L} = \mathcal{L}_{DrugVAE} + \mathcal{L}_{TargetVAE} + \mathcal{L}_{CoVAE}$$

where $\mathcal{L}_{DrugVAE}$ and $\mathcal{L}_{TargetVAE}$ denote the VAE bound for drugs and targets, respectively, and $\mathcal{L}_{CoVAE}$ is a co-regularized term which represents the regression bound responsible for the affinity reconstruction penalty for a pair of drug and target.

Ablation study

To evaluate the robustness and stability of the proposed prediction model, we set four variants for SAGDTI by removing the different modules, namely SAGDTI *I-IV* (see Table S3). Specifically, the SAGDTI *I* is that we removed the molecules embedding module and biological interactive information aggregating module. We add the molecules input embedding module based on the first type, which is set as SAGDTI II. The third type is that we only removed the molecule input embedding module from SAGDTI. We removed the convolutional-pooling layers in the DTI classification module to build the SAGDTI IV. The fifth type is that we do nothing with our proposed model. The results demonstrated that SAGDTI still has state-of-the-art performance when we removed the different modules. Table S4 displays the detailed results of the ablation study.

Model interpretation

SAGDTI includes two main modules: molecular transformer and graph attention network. Both of them are attention-based modules. In order to better analyze what the SAGDTI have learned and what they are based on to make predictions, we analyze the attention weights learned by it, whose visualizations and interpretations can greatly help us to design future models. Based on the attention mechanism in molecular transformer, we can simply understand the underlying mechanism based on the attention weights of compound atoms and protein 3D structures. In the graph attention network, attention coefficients can provide effective explanations of biological networks.

To intuitively illustrate the model interpretability, we conduct a case study, i.e. Replicase polyprotein 1ab (Rep) and remdesivir. We first feed the drug SMILES sequence, protein 3D structure, and biological interaction network into SAGDTI, and then the corresponding attention matrices can be obtained. We use the mean operator to convert three attention matrices into the response attention vectors of remdesivir, Rep, and corresponding interaction information, respectively. Afterward, we map the attention vectors to visualize the interaction between remdesivir and Rep, and the high attention values are highlighted.

As shown in Table S5, the 3D structure of Rep with high attention weights is labeled in light purple, while the sub-structures of drug remdesivir with high attention scores are highlighted in orange in visualization. Furthermore, the biological interaction information extracted by GAT provides guidance to the interaction pattern of the drug-target pair based on the learned attention weights, see top of Table S5.

Figures

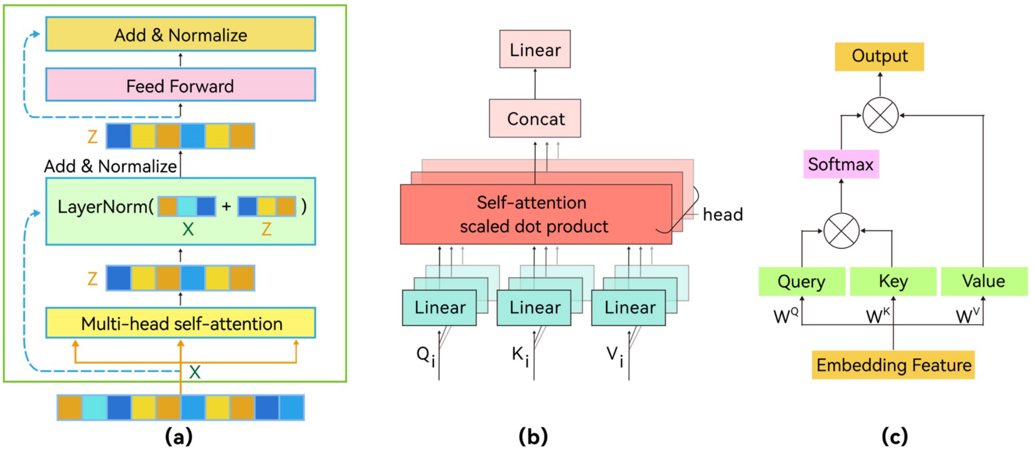

Figure S1. The physical structure of the molecular transformer encoder. (a) Transformer encoder block. (b) Multi-head self-attention. (c) Self-attention scaled dot product.

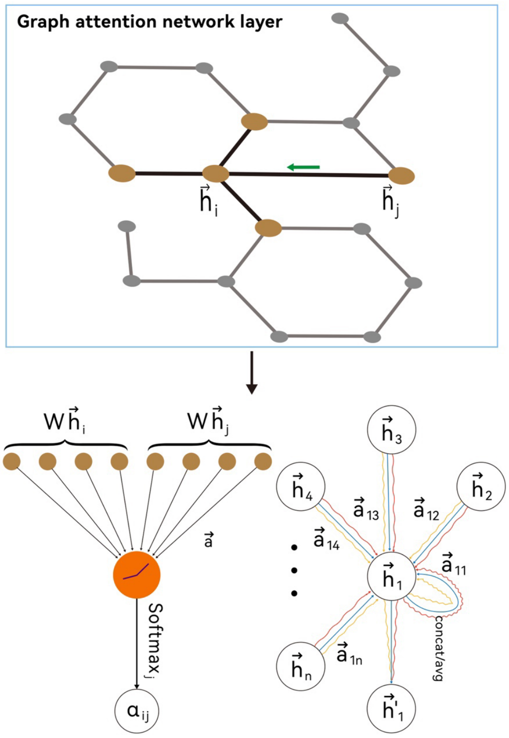

Figure S2. The attention mechanism works between node $\vec{h}_i$ and node $\vec{h}_j$ in a graph attention network layer.

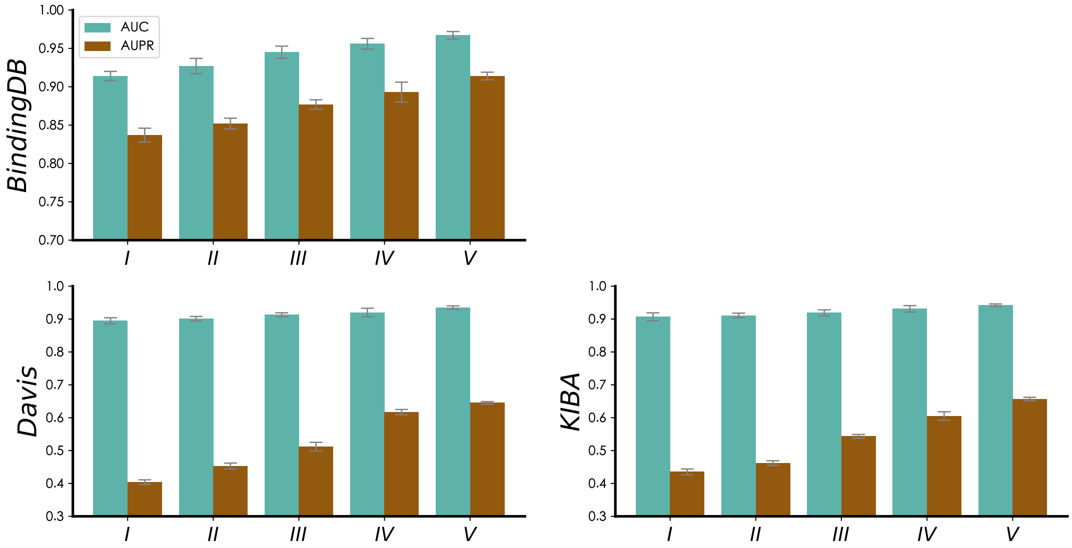

Figure S3. The ablation study on three different datasets.

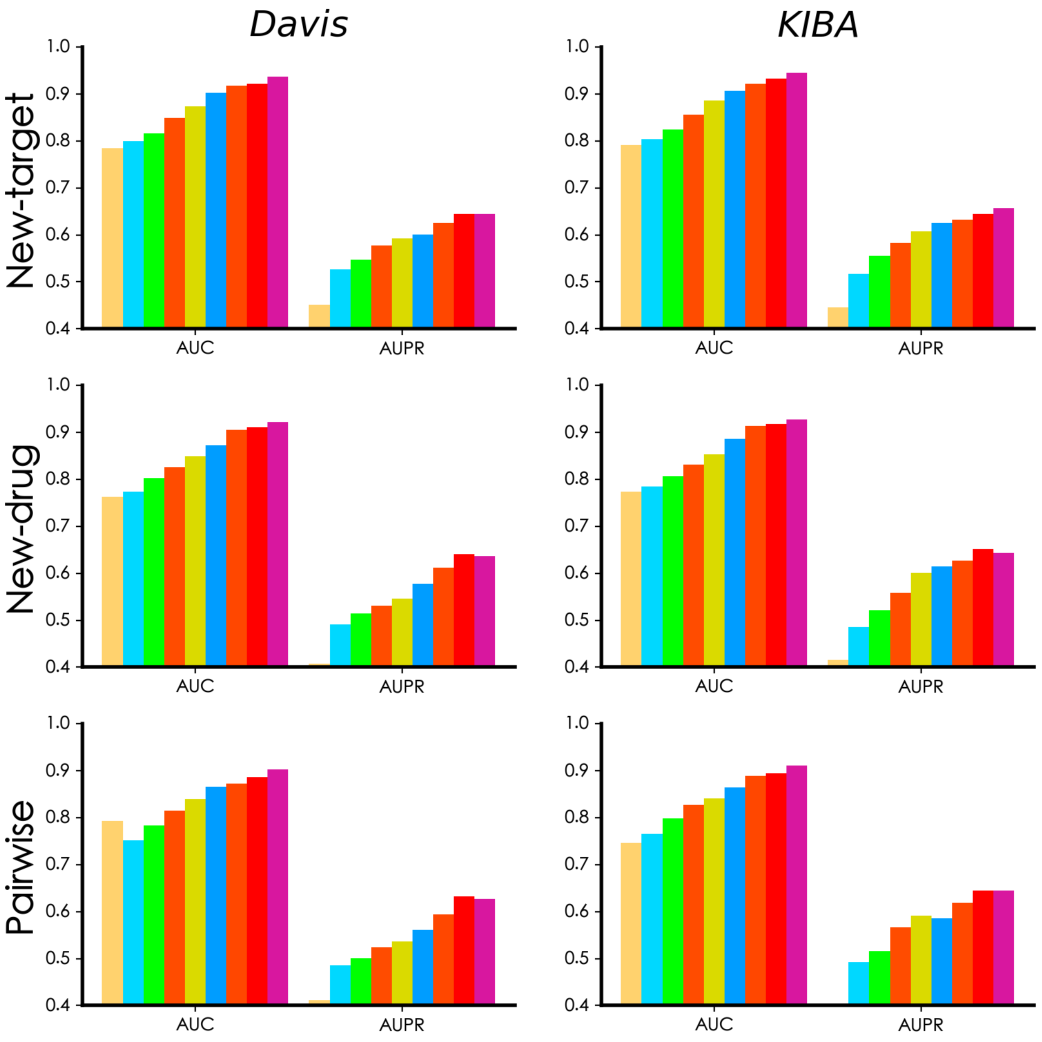

Figure S4. Comparison results of SAGDTI and existing DTI prediction models under three experimental settings on the Davis and KIBA datasets.

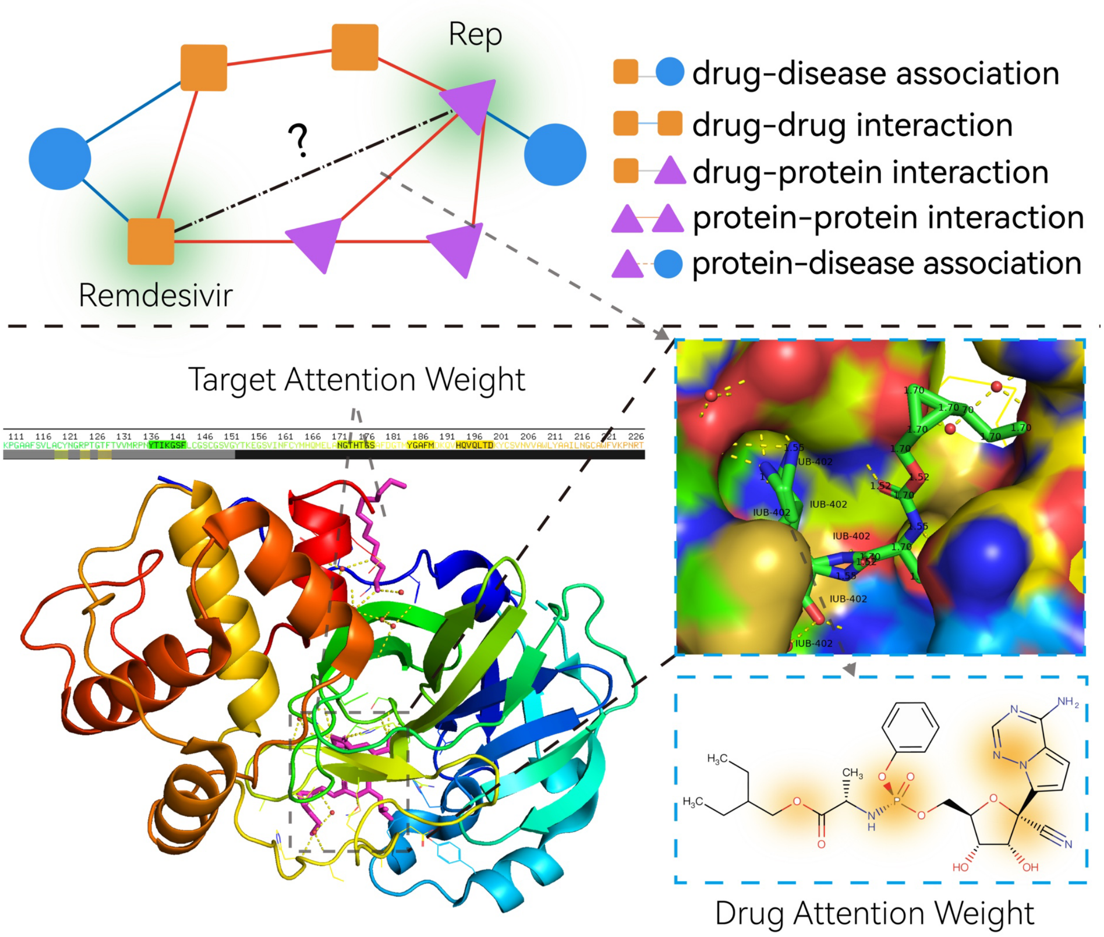

Figure S5. Visualization of attention weights of compounds atom, protein 3D structure, and biological interaction information.

Tables

Table S1. The atom features encoded in each extracted fragment.

Feature Type	Size	Encoding Description
Atom type	11	[C, N, O, S, F, P, Cl, Br, B, H, other] using one-hot encoding
Degree of Atom	6	Number of covalent [0, 1, 2, 3, 4, 5] using one-hot encoding
Radical electrons	1	Number of radical electrons, using an integer to represent
Formal charge	1	Electrical charge, using an integer to represent
Hybrid orbital	6	[sp, sp^2, sp^3, sp^3d, sp^3d^2, other], using one-hot encoding
Is aromatic	1	[0/1], whether the atom belongs to an aromatic system, using one-hot encoding
Hydrogens	5	Number of connected hydrogens [0,1,2,3,4], using one-hot encoding
Chirality	1	[0/1], whether the atom is chiral center, using one-hot encoding
Chirality	2	[S, R] (if none: [0, 0]), using one-hot encoding
Amino acid type	20	The atom on the amino acid, only for receptor, using one-hot encoding

Table S2. Training Hyperparameters of three benchmark datasets (FC is representing the number of fully connected layers, D-MTs and T-MTs are the number of molecular transformer layers for extracting compounds and protein binding sites embedding, respectively, GAT is denoting the number of graph attention network layers for capturing the biological interactive information, and C-P is the number of convolutional-pooling layers for aggregating the molecular and biological attributes.)

Datasets	D-MTs	T-MTs	GAT	C-P	FC
BindingDB	3	5	3	2	2
Davis	5	7	4	3	3
KIBA	5	7	4	3	3

Table S3. Detailed descriptions of the variants of SAGDTI

Variants	Embedding	MTs	GAT	C-P+FC
SAGDTI I	-	✓	-	✓
SAGDTI II	✓	✓	-	✓
SAGDTI II	-	✓	✓	✓
SAGDTI IV	✓	✓	✓	-
SAGDTI	✓	✓	✓	✓

Table S4. The results of the ablation study for five SGADTI types on three benchmark datasets, i.e. BindingDB, Davis and KIBA.

Ablation study	BindingDB		Davis		KIBA	
	AUROC	AUPR	AUROC	AUPR	AUROC	AUPR
SGADTI I	0.914	0.837	0.895	0.404	0.907	0.435
SGADTI II	0.927	0.852	0.901	0.453	0.911	0.462
SGADTI III	0.945	0.877	0.913	0.512	0.919	0.543
SGADTI IV	0.956	0.893	0.920	0.617	0.931	0.605
SGADTI V	0.967	0.914	0.935	0.645	0.942	0.657

Table S5. The ablation study results of different input information on three benchmark datasets.

Input element	BindingDB		Davis		KIBA	
	AUROC	AUPR	AUROC	AUPR	AUROC	AUPR
Without multi-scale interaction	0.912	0.872	0.885	0.613	0.892	0.613
Without drug SMILES sequence	0.943	0.901	0.923	0.625	0.928	0.646
Without protein 3D structure	0.931	0.896	0.904	0.618	0.921	0.637
Without protein 3D structure and multi-scale interaction	0.884	0.849	0.867	0.584	0.878	0.601
Only use multi-scale interaction	0.927	0.883	0.901	0.619	0.914	0.636
Using multi-source information	0.967	0.914	0.937	0.645	0.945	0.657

Table S6. The top 10 candidate proteins predicted by SAGDTI for five drugs against SARS-CoV-2.

Drug	Target	UniPort ID	Rank	Target	UniPort ID	Rank
Remdesivir	Rep	PODTD1	1	CYP2C8	P10632	6
	L	Q05318	2	SLCO1B1	Q9Y6L6	7
	CES1	P23141	3	ABCBI	P08183	8
	CISA	P10619	4	SLCO1B3	Q9NPD5	9
	CYP3A4	P08684	5	ABCC4	015439	10
Lopinavir	pol	Q72874	1	SLCO1B1	Q9Y6L6	6
	CYP3A4	P08684	2	ABCB11	095342	7
	CYP2D6	P10635	3	ALB	P02768	8
	ABCB1	P05177	4	CYP209	P11712	9
	ORMI	P02763	5	CYP2B6	P20813	10
Budesonide	NR3C1	P04150	1	CYP3A4	P08684	6
	CYP3A5	P20815	2	ABCB11	095342	7
	CYP1BI	016678	3	SLCO1A2	P46721	8
	CYP2C8	P10632	4	SLC22A8	Q8TCC7	9
	CYP2C19	P33261	5	SERPINA6	P08185	10
Dexamethasone	NR3C1	P04150	1	CYP3A4	P08684	6
	NROBI	P51843	2	HSD11B1	P28845	7
	ANXA1	P04083	3	CYP3A5	P20815	8
	NOS2	P35228	4	CYP2E1	P05181	9
	HSD11B2	P80365	5	SLCO1A2	P46721	10
Aripiprazole	DRD2	P14416	1	HTR6	P50406	6
	HTR2A	P28223	2	ADRA2B	P18089	7
	ADRA1A	P35348	3	HTRIE	P28566	8
	HTRID	P28221	4	ADRB2	P07550	9
	ADRAZA	P08913	5	CHRM1	P11229	10

Reference

[1] Öztürk H, Özgür A, Ozkirimli E. DeepDTA: deep drug-target binding affinity prediction. *Bioinformatics.* 2018;**34**(17):i821-i829.

[2] Kim Q, Ko JH, Kim S, et al. Bayesian neural network with pretrained protein embedding enhances prediction accuracy of drug-protein interaction. *Bioinformatics.* 2021;**37**(20):3428-3435.

[3] Gao KY, Fokoue A, Luo H, et al. Interpretable drug tar-get prediction using deep neural representation. IJCAI 2018; 3371–7.

[4] Chu Y, Kaushik AC, Wang X, et al. DTI-CDF: a cascade deep forest model towards the prediction of drug-target interactions based on hybrid features. *Brief Bioinform.* 2021;**22**(1):451-462.

[5] Zhao Q, Zhao H, Zheng K, et al. HyperAttentionDTI: improving drug-protein interaction prediction by sequence-based deep learning with attention mechanism. *Bioinformatics.* 2022;**38**(3):655-662.

[6] Zeng Y, Chen X, Luo Y, et al. Deep drug-target binding affinity prediction with multiple attention blocks. *Brief Bioinform.* 2021;**22**(5):bbab117.

[7] Wallach I, Dzamba M, Heifets A. Atomnet: a deep convolutional neural network for bioactivity prediction in structure-based drug discoveryarXiv preprint arXiv:1510.02855. 2015.

[8] Li T, Zhao XM, Li L. Co-VAE: Drug-Target Binding Affinity Prediction by Co-Regularized Variational Autoencoders. *IEEE Trans Pattern Anal Mach Intell.* 2022;**44**(12):8861-8873.

[9] Karimi M, Wu D, Wang Z, et al. DeepAffinity: interpretable deep learning of compound-protein affinity through unified recurrent and convolutional neural networks. *Bioinformatics.* 2019;**35**(18):3329-3338.

[10] Nguyen T, Le H, Quinn TP, et al, Venkatesh S. GraphDTA: predicting drug-target binding affinity with graph neural networks. *Bioinformatics.* 2021;**37**(8):1140-1147.

[11] Xuan P, Zhang X, Zhang Y, Hu K, Nakaguchi T, Zhang T. Multi-type neighbors enhanced global topology and pairwise attribute learning for drug-protein interaction prediction. *Brief Bioinform.* 2022;**23**(5):bbac120.

[12] Hu K, Cui H, Zhang T, et al. ALDPI: adaptively learning importance of multi-scale topologies and multi-modality similarities for drug-protein interaction prediction. *Brief Bioinform.* 2022;**23**(2):bbab606.

[13] Peng J, Wang Y, Guan J, et al. An end-to-end heterogeneous graph representation learning-based framework for drug-target interaction prediction. *Brief Bioinform.* 2021;**22**(5):bbaa430.

[14] Zhao T, Hu Y, Valsdottir LR, et al. Identifying drug-target interactions based on graph convolutional network and deep neural network. *Brief Bioinform.* 2021;**22**(2):2141-2150.

[15] Yazdani-Jahromi M, Yousefi N, Tayebi A, et al. AttentionSiteDTI: an interpretable graph-based model for drug-target interaction prediction using NLP sentence-level relation classification. *Brief Bioinform.* 2022;**23**(4):bbac272.

[16] Torng W, Altman RB. Graph Convolutional Neural Networks for Predicting Drug-Target Interactions. *J Chem Inf Model.* 2019;**59**(10):4131-4149.

[17] Mohamed SK, Nováček V, Nounu A. Discovering protein drug targets using knowledge graph embeddings. *Bioinformatics.* 2020;**36**(2):603-610.

[18] Gilson MK, Liu T, Baitaluk M, et al. BindingDB in 2015: A public database for medicinal chemistry, computational chemistry and systems pharmacology. *Nucleic Acids Res.* 2016;**44**(D1):D1045-D1053.

[19] Davis AP, Murphy CG, Johnson R, et al. The Comparative Toxicogenomics Database: update 2013. *Nucleic Acids Res.* 2013;**41**:D1104-D1114.

[20] Tang J, Szwajda A, Shakyawar S, et al. Making sense of large-scale kinase inhibitor bioactivity data sets: a comparative and integrative analysis. *J Chem Inf Model.* 2014;**54**(3):735-743.

[21] Kim S, Chen J, Cheng T, et al. PubChem in 2021: new data content and improved web interfaces. *Nucleic Acids Res.* 2021;**49**(D1):D1388-D1395.

[22] Berman HM, Westbrook J, Feng Z, et al. The Protein Data Bank. *Nucleic Acids Res.* 2000;**28**(1):235-242.

[23] Peng J, Wang Y, Guan J, et al. An end-to-end heterogeneous graph representation learning-based framework for drug-target interaction prediction. *Brief Bioinform.* 2021;**22**(5):bbaa430.

[24] He T, Heidemeyer M, Ban F, et al. SimBoost: a read-across approach for predicting drug-target binding affinities using gradient boosting machines. *J Cheminform.* 2017;**9**(1):24.

[25] Olayan RS, Ashoor H, Bajic VB. DDR: efficient computational method to predict drug-target interactions using graph mining and machine learning approaches. *Bioinformatics.* 2018;**34**(7):1164-1173.

[26] Liu S, Wang Y, Deng Y, et al. Improved drug-target interaction prediction with intermolecular graph transformer. *Brief Bioinform.* 2022;**23**(5):bbac162.

[27] Huang K, Xiao C, Glass LM, et al. MolTrans: Molecular Interaction Transformer for drug-target interaction prediction. *Bioinformatics.* 2021;**37**(6):830-836.
